# CALB2 mediates tumor progression and immune escape in colorectal cancer by shaping an inhibitory immune microenvironment

**DOI:** 10.3389/fimmu.2026.1791363

**Published:** 2026-04-15

**Authors:** Yibo Bian, Chaonan Huangfu, Yuanyuan Lu

**Affiliations:** 1 State Key Laboratory of Holistic Integrative Management of Gastrointestinal Cancers, National Clinical Research Center for Digestive Diseases, Xijing Hospital of Digestive Diseases, Xijing Hospital, Fourth Military Medical University, Xi’an, Shaanxi, China; 2 Department of Oncology, BenQ Hospital of Nanjing Medical University, Nanjing, Jiangsu, China

**Keywords:** CALB2, cancer-associated fibroblast, CD8^+^T cell, immune microenvironment, macrophage

## Abstract

**Background:**

Colorectal cancer (CRC) remains a leading cause of cancer-related mortality worldwide, characterized by a highly immunosuppressive tumor microenvironment (TME) that facilitates immune evasion and tumor progression.

**Methods:**

Integrated analysis of TCGA and GEO datasets was performed to evaluate CALB2 expression and its prognostic significance in CRC. Functional experiments including proliferation, migration, colony formation, and apoptosis assays were conducted following CALB2 knockdown. Co-culture assays and multiplex immunohistochemistry (mIHC) were used to assess TME remodeling.

**Results:**

Elevated CALB2 expression correlated significantly with poor prognosis in CRC patients. CALB2 knockdown suppressed CRC cell proliferation, migration, and colony formation, while promoting apoptosis. CALB2 silencing also reprogrammed TME by inducing macrophage M1 polarization, enhancing CD8^+^T cell cytotoxicity, and reducing immunosuppressive cell infiltration. Mechanistically, CALB2 activated the STAT3 signaling pathway to promote CCL5 secretion, facilitating M2 polarization of macrophages and activation of fibroblasts.

**Conclusion:**

Our findings identify CALB2 as a critical regulator of tumor progression and immune escape in CRC, acting through dual oncogenic and immunomodulatory mechanisms. CALB2 represents a promising therapeutic target for enhancing immunotherapy efficacy in CRC.

## Introduction

Colorectal cancer (CRC) remains a leading cause of cancer-related morbidity and mortality worldwide, presenting a significant public health challenge (1). Recent epidemiological data indicated that CRC ranks third in incidence and second in mortality globally, with an estimated 2.2 million new cases and 1.0 million deaths (2–4). Given the persistent challenges in clinical management, the identification of novel biomarkers for early detection is essential to reduce mortality and improve prognosis for CRC patients.

The tumor immune microenvironment plays a critical role in the progression of tumors and closely correlates with treatment responses (5). M2 macrophages can secrete immunosuppressive cytokines to inhibit the anti-tumor activity of other immune cells, while promoting angiogenesis and tissue repair, thereby creating favorable conditions for tumor growth, invasion and metastasis (6). CD8^+^T cells can recognize and eliminate tumor cells, yet their functions are often inhibited (7). Activated cancer-associated fibroblasts (CAFs) can secrete a large amount of extracellular matrix and signaling molecules, forming a physical and chemical barrier (8). This not only hinders the infiltration of immune cells such as CD8^+^T cells into the tumor interior but also actively recruits M2 macrophages, jointly creating an immunosuppressive environment (9). Therefore, reversing this immunosuppressive state has become an important strategy in current cancer immunotherapy.

Calbindin 2, encoded by the CALB2 gene, is a calcium-binding protein that functions as a calcium buffer and signal sensor in the cell interior (10). CALB2 has been reported to be associated with the staging of lung cancer, ovarian cancer, pancreatic cancer, and malignant mesothelioma (11, 12). Recent studies have revealed that CALB2 can regulate the function of CAF in the immune microenvironment of pancreatic cancer (13). Nevertheless, the biological function of CALB2 in CRC remains unclear.

Based on these observations, this study aims to investigate the role of CALB2 in CRC and to identify CALB2 as a potential biomarker in the immune microenvironment. Using TCGA database, clinical samples, and functional assays, we found that CALB2 overexpression is associated with poor prognosis and suppressive immune microenvironment. Knockdown of CALB2 inhibits cell proliferation, migration, M2 macrophage polarization and increases CD8^+^T cell cytotoxicity. CALB2 promotes CCL5 secretion through STAT3 pathway activation, which subsequently induces M2 macrophage polarization and fibroblast activation. These findings offer novel insights into CRC progression and suggest CALB2 as a potential therapeutic target.

## Materials and methods

### Databases

The data of CRC patients were retained from the TCGA database (https://portal.gdc.cancer.gov). The prognostic value of CALB2 from TCGA-CRC and GEO cohorts was analyzed via GEPIA website (http://gepia.cancer-pku.cn/) and BEST sites (https://best.com/analysis/), including overall survival (OS) and disease-free survival (DFS). RNA-seq data from the GEO project were analyzed via GEO2R website and BEST sites (https://best.com/analysis/). Immune infiltration analysis on the TCGA and GEO datasets was conducted through the CIBERSORT website and the BEST website. Gene expression comparisons and Kaplan-Meier survival analysis were performed using *R* software.

### Cell culture

CRC cell lines (HCT116, HT-29, SW480, SW620, DLD-1, and HCT8), and normal human colonic mucosal cell line (NCM460) were maintained from Sciences cell bank of Chinese Academy. All cell lines were cultured in DMEM or RPMI1640 containing 10% fetal bovine serum (Gemini, USA), 100 mg/mL streptomycin and 100 U/mL penicillin. All cells were routinely cultured in a cell incubator at 37 °C containing 5% CO2, and it was routinely confirmed that there was no mycoplasma contamination.

### Plasmid construction and cell transfection

CALB2-targeting small interference RNAs (siRNA) and CALB2 overexpression plasmid were designed and manufactured by Haixing Biosciences (Suzhou, China). According to the instructions, transfect the small interference and plasmid into the cells with lipo2000 (Invitrogen, USA). In summary, lipo2000, plasmids and siRNAs were dissolved in optimum for 5 min, then gently mixed and incubated at room temperature for 20 min, added to cells, and changed medium 8–12 h later.

### RNA extraction and RT-qPCR

Total RNA from cells were extracted by Trizol reagent (Invitrogen, USA) and reversely transcribed into cDNA. Then based on the manufacturer’s instructions, RT-qPCR was performed by SYBR Green Master (Vazyme, China) using cDNA as template. The comparative threshold cycle (2-^ΔΔCt^) method was used to analyze the expression of related genes, and GAPDH was used as the internal reference. All used primers were demonstrated in **Supplementary Table 1**.

### Western blot

Protein from treated cells were extracted with RIPA (Beyotime, China) and quantified by BCA Assay Kit (Beyotime, China). 20 μg total protein was separated by SDS-PAGE and then transferred onto PVDF membranes. The membranes were blocked with 5% skim milk for 1 h at room temperature and then incubated at 4 °C overnight with specific antibodies CALB2 (Proteintech, China), Slug (Proteintech, China), Snail1 (Proteintech, China), GAPDH (Proteintech, China) and β-actin (Proteintech, China). On the second day, the second antibody (Proteintech, China) was incubated with the membrane at room temperature for 1 h and then membrane was visualized by ECL substrate (Vazyme, China) after three washes with TBST. The intensity of immunoreactive bands were quantified by the ImageJ software and normalized to β-actin or GAPDH levels.

### Cell proliferation and colony formation assays

Both CCK8 assay and colony formation assay were used to detect the cell proliferation ability. 2000–4000 treated tumor cells were seeded into 96-well plates and CCK8 solution was added every 24 h to detect absorbance at 450 nm. In colony formation, 500–1000 treated tumor cells were seeded in 6- well plates and incubated at 37 °C for 14 days. Then cells were fixed with methanol and stained with 0.1% crystal violet. The number of forming colonies were pictured and analyzed.

### EdU assays

EdU (5-Ethynyl-2’-deoxyuridine) staining is another cell proliferation assay, which is used to label and detect cells in the DNA synthesis phase (S phase) to reflect cell proliferation. Briefly, cells were inoculated in 24-well plates and experiment was conducted 48 h after transfection. Dilute EdU to 20 µM, add 100 µL of EdU working solution to each well and continue to incubate at 37 °C for 2 h to ensure that EdU is fully incorporated into the newly synthesized DNA. Discard the culture medium and wash with PBS. Fix the cells with 4% paraformaldehyde for 30 min. Add 50 µL of the reaction solution to each well and incubate at room temperature in the dark for 30 min to ensure that the fluorescent dye fully combines with EdU. Finally, the cell nuclei were stained with DAPI and incubated in the dark for 5–10 min. Images were collected using fluorescence microscope or confocal microscope and quantitative analysis of cell proliferation was conducted. The proliferation of cells is evaluated by counting the number of EdU positive cells.

### Migration assay

Cell migration assays were performed using 24-well transwell chambers with 8 μm pore polycarbonate membrane inserts (Millipore, USA). The lower chamber of insert was added medium which supplied with 20% fetal bovine serum, 5×10^4^ transfected tumor cells in serum-free medium were added to the upper chamber of insert (Millipore, USA) and then incubated about 24–48 h. Cells passing through the membrane were fixed with 4% paraformaldehyde and then stained with 0.1% crystal violet.

### Cell apoptosis detection

Treated tumor cells were collected after 48 h of transfection and resuspended within Binding buffer, then mixed with fluorescently labeled AnnexinV-FITC and PI Staining Solution (Vazyme, China) for 15 min in dark room. Then the apoptosis state was detected via flow cytometer (Beckman Coulter, USA) and the percentage of apoptosis was analyzed with flowJo software.

### Cytokine detection

PBMCs (Peripheral Blood Mononuclear Cells) is extracted from the blood of healthy donors using Ficoll-plaque separation media and activated with CD3/28 antibodies, and then cultured in 1640 medium containing IL2 (Miltenyi Biotec, Germany). Transfected tumor cells and PBMCs were co-cultured for 24–48 h before cytokine assays. Brefeldin A (Biolegend, USA) was added to PBMCs and then incubated at 37 °C for 3–4 h to block cytokines within the cells. Wash 1–2 times with PBS and then stain with Zombie NIR (ABclonal, China) at 4 °C in the dark for 10 min. Then stain surface marker including CD45 (Miltenyi Biotec, Germany) and CD8 (Miltenyi Biotec, Germany) at 4 °C in the dark for 20 min after 1–2 times with PBS. The cells were then fixed at room temperature for 30 min. After washing with permeabilization wash buffer 1–2 times, TNFα, GzmB and IFNγ (ABclonal, China) were stained in the dark for 20 min. Finally, cells were dissolved in PBS for flow cytometry (Beckman Coulter, USA).

### T cell killing assay

Tumor cell viability following co-culture with pre-activated PBMCs was assessed using a Cell Counting Kit-8 (GlpBio, USA) assay. Briefly, target tumor cells were seeded in 96-well plates and allowed to adhere overnight. Activated PBMCs were then added at the indicated effector-to-target ratios. After co-incubation, non-adherent cells were removed by washing, and the viability of the adherent tumor cells was measured by CCK-8 assay per the manufacturer’s protocol, with absorbance read at 450 nm. Cell viability was normalized to control tumor cells cultured in the absence of PBMCs.

### LDH release assay

Cytotoxicity was assessed using a lactate dehydrogenase (LDH) release assay. Pre-activated PBMCs were co-cultured with target tumor cells in 96-well plates at indicated effector-to-target (E: T) ratios. After 4h of incubation, the supernatant was collected, and LDH release was measured according to the manufacturer’s instructions (GlpBio, China). The percentage of cytotoxicity was calculated relative to the maximum LDH release control.

### Immunofluorescence staining

For immunofluorescence staining, formalin-fixed paraffin-embedded (FFPE) tissue sections were deparaffinized in xylene and rehydrated through graded ethanol. Antigen retrieval was performed by heating the sections in citrate buffer at 95 °C for 20 min. After cooling, sections were permeabilized with 0.3% Triton X-100 in PBS for 15 min and blocked with 5% normal goat serum in PBST for 1 h at room temperature. The sections were then incubated with primary antibody overnight at 4 °C, followed by incubation with fluorophore-conjugated secondary antibody for 1 h at room temperature in the dark. Nuclei were counterstained with DAPI for 15 min. Images were captured using a fluorescence microscope (Olympus, Japan) and analyzed with ImageJ software.

### Multiplex immunohistochemistry

Paraffin sections of the patient’s tissues were dewaxed and hydrated in sequence with xylene, anhydrous ethanol, 95% ethanol, 85% ethanol, and 75% ethanol, and then antigen restoration was carried out using alkaline EDTA or citric acid buffer. After blocking for 30 min, the primary antibody was incubated for 1 h at room temperature followed by 30 min of secondary antibody incubation, dye (ImmunoWay, USA) was incubated in the dark for 15 min. Wash 3 times with PBST for antigen repair, and the above process was repeated until all indicators were stained. DAPI was incubated at room temperature for 10min, then washed with PBST for 3 times. Anti-fluorescence quench sealant was added drip to seal tablets, and finally scanned for imaging and data analysis.

### Statistical analysis

Univariate and multivariate Cox proportional hazards regression models were used to identify independent prognostic factors. Hazard ratios (HRs) and 95% confidence intervals (CIs) were calculated. All statistical tests were two-sided, and *P* < 0.05 was considered statistically significant. Analysis was performed using SPSS version 26.0. GraphPad Prism version 10.0, and Image J were used for data analysis and graphing. All experiments were independently repeated three times and presented as the mean ± standard deviation (SD). Data difference between two groups were analyzed by Student’s t-test while ANOVA was used to compare more than two groups. Survival curves were analyzed using the Kaplan-Meier method and compared using the log-rank test. Significant differences are represented as *P < 0.05, **P < 0.01, ***P < 0.001.

## Results

### CALB2 is upregulated in CRC tissues and correlated with poor prognosis

**Figure 1:**
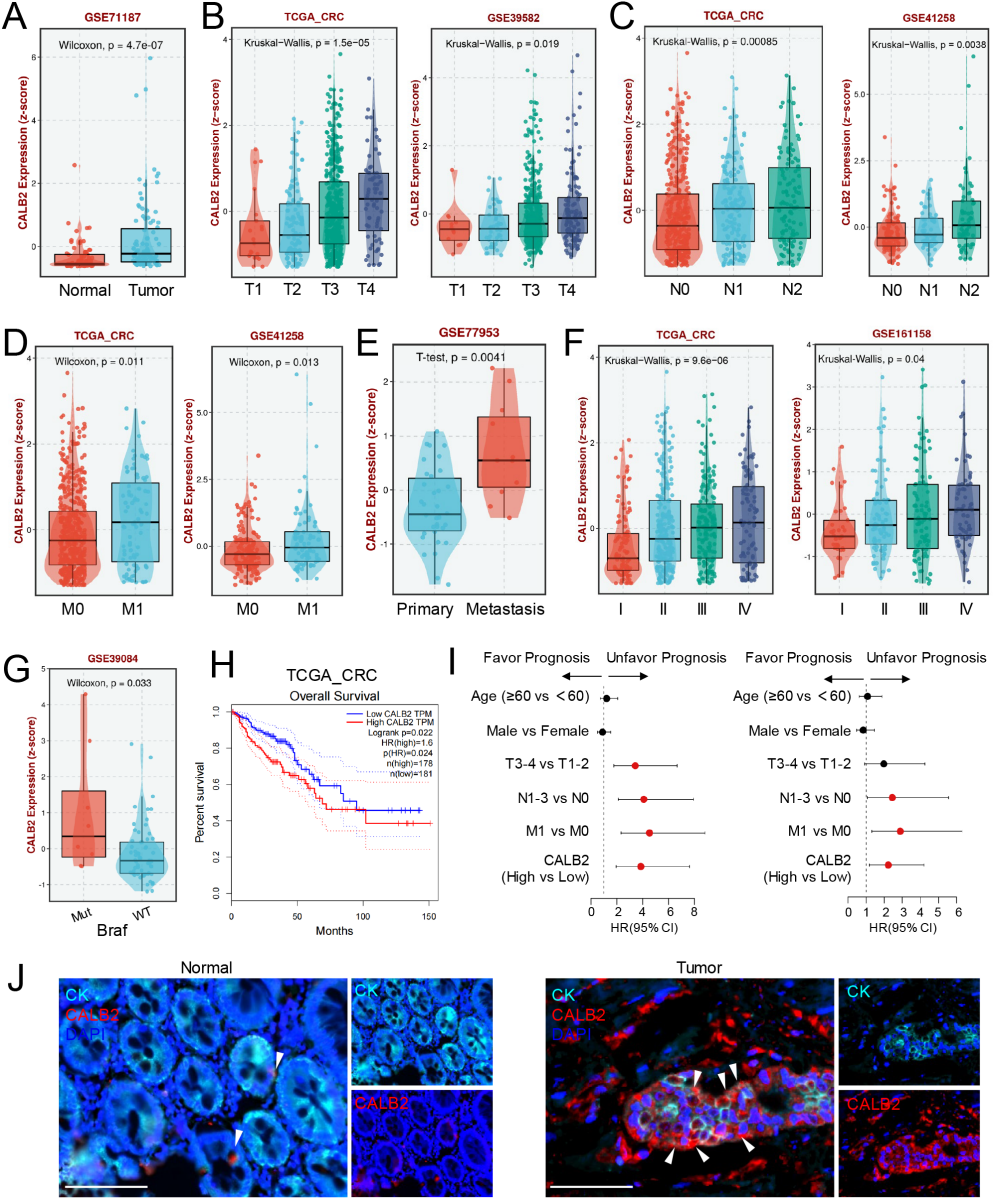
CALB2 is upregulated in CRC tissues and correlated with poor prognosis. **(A)** The expression of CALB2 in normal and tumor tissues from GSE71187 cohort. **(B)** The expression of CALB2 in different primary tumor stages from TCGA-CRC and GSE39582 cohorts. **(C)** The expression of CALB2 in different lymph node invasion stages from TCGA-CRC and GSE41258 cohorts. **(D)** The expression of CALB2 in metastatic and non-metastatic tissues from TCGA-CRC and GSE41258 cohorts. **(E)** The expression of CALB2 in primary and metastatic tissues from GSE77953 cohort. **(F)** The expression of CALB2 at different clinical stages from TCGA-CRC and GSE161158 cohort. **(G)** The expression of CALB2 in *Braf* mutant and wild-type patients from GSE39084 cohort. **(H)** Overall survival analysis of colorectal cancer patients with high and low CALB2 expression from TCGA-CRC cohort. **(I)** Forest plot of hazard ratios (HRs) for overall survival (OS) according to pathological characteristics. Univariate (left) and multivariate (right) Cox regression analysis were performed on our patient cohort. Red indicates significant differences and black shows no significant differences. **(J)** Representative immunofluorescence images of normal adjacent tissue and tumor tissues. The cell nuclei are labeled by DAPI, CALB2 is labeled by anti-CALB2 antibody and tumor epithelial cells are labeled by anti-CK antibody. Scale: 50 μm. Scar bar:50 μm.

To investigate the clinical significance of CALB2 in CRC, we analyzed its expression in public datasets and our own cohort. Analysis of the GSE71187 dataset revealed that CALB2 was highly expressed in colorectal tumor tissue (**Figure 1A**). In the TCGA-CRC, GSE39582, and GSE41258 cohorts, CALB2 expression was higher in T4 stage compared with T1 stage (**Figure 1B**; **Supplementary Figure S1A**). Similarly, CALB2 was highly expressed in N2 stage compared with N0 stage in TCGA-CRC and GSE41258 cohorts (**Figure 1C**). Data from TCGA-CRC and GSE41258 cohorts also showed CALB2 high expression in M1 stage (**Figure 1D**). CALB2 was also highly expressed in metastasis tissue from GSE77953 cohort (**Figure 1E**) and CALB2 expression was higher in stage IV compared with stage I in TCGA-CRC, GSE161158 and GSE38832 cohorts (**Figure 1F**; **Supplementary Figure S1B**). Additionally, CALB2 was also highly expressed in *BRAF* Mut patients from GSE39084 cohort (**Figure 1G**). Meanwhile, high expression of CALB2 was associated with poor overall survival from TCGA-CRC and GSE17537 cohorts (**Figure 1H**; **Supplementary Figure S1C**), additionally, Kaplan-Meier analysis showed that patients with high CALB2 expression had shorter disease-free survival and shorter progress-free survival than those with low CALB2 expression (**Supplementary Figures S1D, E**). CALB2 expression was positively associated with invasion depth, and lymph node state and metastasis in our cohort (**Table 1**). Univariate and multivariate Cox regression analyses further revealed that CALB2 expression was an independent prognostic factor for predicting outcome in CRC patients based on our cohort (**Figure 1I**; **Table 2**). Then we detected CALB2 from our team CRC tissues via Immunofluorescence, the result showed that CALB2 was high expressed in tumor tissues contrast with adjacent normal tissues (**Figure 1J**). In summary, high expression of CALB2 was associated with unfavorable prognosis in CRC patients.

**Table 1:** Correlation between CALB2 expression and clinicopathological characteristics of 36 CRC patients.

Variables	Total	Low CALB2 group	High CALB2 group	*P* value
	patients			chi-squared test
All cases	36	18	18	
Age				0.737
≤60	16	9	7	
>60	20	9	11	
Sex				0.737
male	20	9	11	
female	16	9	7	
Tumor				0.0045 **
T1 andT2	13	11	2	
T3 and T4	23	7	16	
Lymph node				<0.001 **
N0	16	14	2	
N1/2/3	20	4	16	
Distant metastasis				0.0408 *
Negative	28	17	11	
Positive	8	1	7	

* *p* < 0.05, ** *p* < 0.01.

**Table 2:** Univariate and multivariate Cox regression analysis of overall survival in CRC patients.

Variable	Univariate analysis	p-value	Multivariate analysis	p-value
	HR (95% CI)		HR (95% CI)	
CALB2 expression (High vs Low)	3.85 (1.95-7.60)	<0.001	2.23 (1.18-4.21)	0.013
T stage (T3-T4 vs T1-T2)	3.42 (1.76-6.65)	<0.001	1.98 (0.92-4.26)	0.081
N stage (N1-N2 vs N0)	4.08 (2.10-7.93)	<0.001	2.45 (1.08-5.56)	0.032
M stage (M1 vs M0)	4.52 (2.32-8.81)	<0.001	1.98 (0.87-4.51)	0.008
Age (continuous)	1.21 (0.71-2.06)	0.485	1.08 (0.62-1.88)	0.782
Gender (Male vs Female)	0.89 (0.52-1.52)	0.668	0.84 (0.48-1.47)	0.541

### CALB2 promotes cell proliferation and inhibits apoptosis of CRC *in vitro*

**Figure 2:**
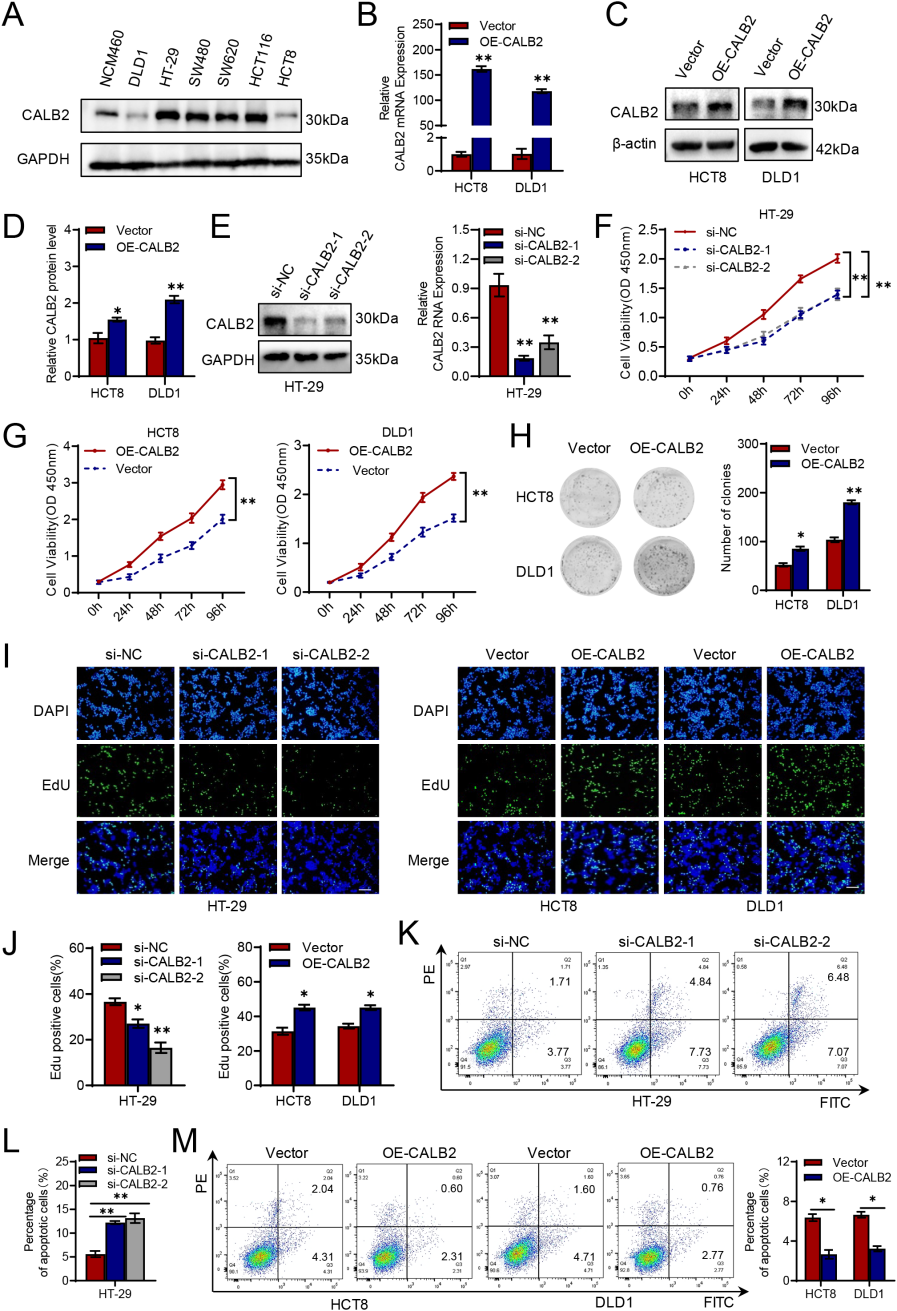
CALB2 promotes cell proliferation and inhibits apoptosis of CRC *in vitro*. **(A)** Immunoblotting (IB) analysis of CALB2 in multiple colorectal cancer cell lines. **(B)** The level of CALB2 mRNA after transfecting CALB2 plasmid was detected by quantitative reverse transcription PCR (RT-qPCR). **(C)** Immunoblotting (IB) analysis of CALB2 after transfecting CALB2 plasmid. **(D)** Quantitative analysis of CALB2 protein expression after transfecting CALB2 plasmid. **(E)** Immunoblotting (IB) analysis of CALB2 and the level of CALB2 mRNA after transfecting si-CALB2. **(F)** Viability of HT-29 cells transfected with si-CALB2 was detected by CCK8 assay. **(G)** Viability of DLD1 and HCT8 cells transfected with CALB2 plasmid was detected by CCK8 assay. **(H)** Viability of DLD1 and HCT8 cells transfected with CALB2 plasmid was detected by clone formation assay. **(I)** Viability of HT-29 cells transfected with si-CALB2, DLD1 and HCT8 cells transfected with CALB2 plasmid were detected by EdU assay. Scar bar:200 μm. **(J)** Analysis of the proportion of EdU positive cells. **(K, L)** The proportion of apoptotic cells in HT-29 cells transfected with si-CALB2 was detected by flow cytometry. **(M)** The proportion of apoptotic cells in HCT8 and DLD1 cells transfected with CALB2 plasmid was detected by flow cytometry. *P < 0.05, **P < 0.01.

Firstly, we detected the expression of CALB2 in colorectal cancer cell lines through western blot and the result revealed that CALB2 was upregulated in HT-29, SW480, SW620 and HCT116 cell lines comparing with the human normal colon epithelial cell (NCM460) (**Figure 2A**). Then, we chose HCT8, DLD1 and HT-29 cell lines for further study. Firstly, we designed over-expressed CALB2 plasmid and two siRNAs to explore its biological role in CRC. We detected CALB2 expression via RT-qPCR and western blot after transfecting plasmid, the results showed CALB2 was high expressed in both mRNA (**Figure 2B**) and protein level (**Figures 2C, D**), indicating the efficiency of plasmids. Similarly, efficiency of siRNA targeting CALB2 was confirmed valid via western blot and RT-qPCR (**Figure 2E**). The proliferation ability of CALB2 was detected via CCK8, colony formation and EdU assays. Silencing CALB2 significantly suppressed cell viability compared with negative control group in HT-29 cell lines (**Figure 2F**). Meanwhile, over-expressing CALB2 facilitated cell proliferation in HCT8 and DLD1 cell lines (**Figure 2G**). Similarly, the number of cell colonies increased when over expressing CALB2 in HCT8 and DLD1 cell lines (**Figure 2H**). CALB2 knockdown decreased cell colonies in HT-29 cell (**Supplementary Figure S1F**). We obtained similar results in the EdU assays. Silencing CALB2 significantly reduced the proportion of proliferating cells in HT-29 cell while elevated CALB2 remarkably improve the cell proliferation in HCT8 and DLD1 cells (**Figures 2I, J**). Flow cytometry was applied to evaluate the effect of CALB2 in apoptosis. Our results revealed that the fraction of apoptotic cells was increased significantly when silencing CALB2 in HT-29 cell (**Figures 2K, L**), and over expressing CALB2 decreased the portion of apoptosis in HCT8 and DLD1 cells (**Figure 2M**).

### CALB2 promotes cell migration of CRC *in vitro*

**Figure 3:**
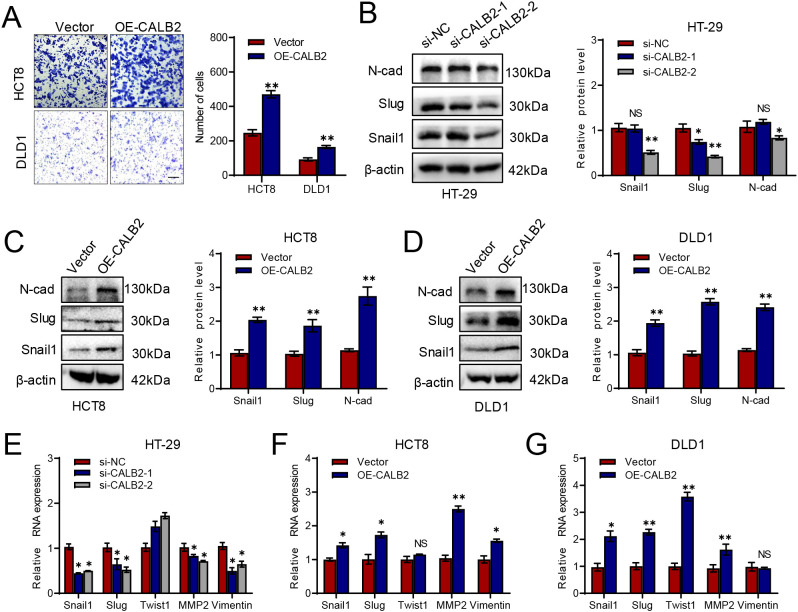
CALB2 promotes cell migration of CRC *in vitro*. **(A)** The migration ability of DLD1 and HCT8 cells transfected with CALB2 plasmid was detected by transwell assay. **(B)** Immunoblotting (IB) analysis and quantitative analysis of Slug, Snail1 and N-cadherin in HT-29 cells transfected with si-CALB2. **(C)** Immunoblotting (IB) analysis and quantitative analysis of Slug, Snail1 and N-cadherin in HCT8 cells transfected with CALB2 plasmid. **(D)** Immunoblotting (IB) analysis and quantitative analysis of Slug, Snail1 and N-cadherin in DLD1 cell transfected with CALB2 plasmid. **(E)** The mRNA levels of EMT-related genes (Snail1, Slug, Twist1, MMP2, and Vimentin) in HT-29 cells transfected with si-CALB2 were detected by RT-qPCR. **(F)** The mRNA levels of EMT-related genes (Snail1, Slug, Twist1, MMP2, and Vimentin) in HCT8 cells transfected with CALB2 plasmid were detected by RT-qPCR. **(G)** The mRNA levels of EMT-related genes (Snail1, Slug, Twist1, MMP2, and Vimentin) in DLD1 cells transfected with CALB2 plasmid were detected by RT-qPCR. *P < 0.05, **P < 0.01.

We performed transwell assay to observe the migration change and the result revealed overexpressing CALB2 augmented migration ability in HCT8 and DLD1 cells (**Figure 3A**). Next, we detected epithelial-mesenchymal transition (EMT)-related proteins such as N-cadherin, Slug and Snail1 via western blot. Silencing CALB2 suppressed N-cadherin, Slug and Snail1 expression, especially they were all decreased significantly when transfected si-CALB2–2 in HT-29 cells (**Figure 3B**). Overexpression of CALB2 increased expression of N-cadherin, Slug and Snail1 in HCT8 cells (**Figure 3C**) and the similar result was also observed in DLD1 cells (**Figure 3D**). Snail and Slug showed consistent upregulation at both mRNA and protein levels upon CALB2 overexpression, indicating that these transcription factors may mediate the subsequent induction of epithelial-mesenchymal transition (EMT) (**Figures 3F, G**). Overexpression or knockdown of CALB2 led to inconsistent changes in the mRNA levels of other EMT related genes (Twist1 and Vimentin) (**Figures 3E–G**). This discrepancy may be attributed to post transcriptional regulation of these mRNAs or to a less pivotal role of these genes in the CALB2 driven EMT process. Taken together, our findings indicated that CALB2 enhances the migratory ability of colorectal cancer cells.

### Immune infiltration analysis of CALB2 in colorectal cancer

**Figure 4:**
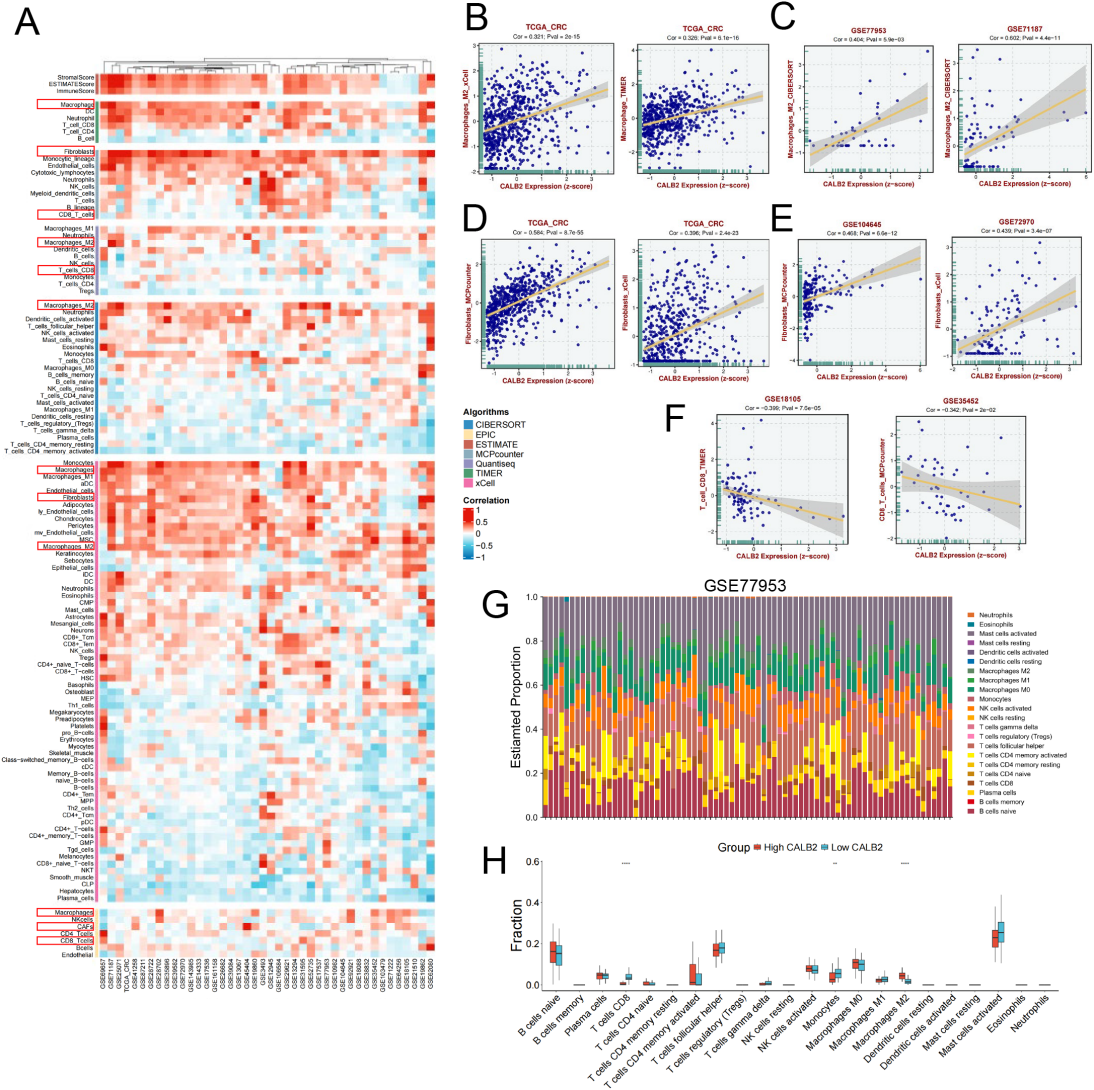
Immune infiltration analysis of CALB2 in colorectal cancer. **(A)** Heat maps of correlation analysis of CALB2 immune infiltration in GEO datasets and TCGA dataset from different algorithms (CIBERSORT, EPIC, ESTIMATE, MCPcounter, Quantiseq, TIMER and xCELL algorithms). **(B)** Correlation analysis between CALB2 and macrophages from TCGA cohorts. **(C)** Correlation analysis between CALB2 and macrophages from GSE77953 and GSE71187 cohorts. **(D)** Correlation analysis between CALB2 and fibroblasts from TCGA dataset. **(E)** Correlation analysis between CALB2 and fibroblasts from GSE104645 and GSE72970 cohorts. **(F)** Correlation analysis between CALB2 and CD8^+^T cell from GSE18105 and GSE35452 cohorts. **(G)** Analysis charts of immune cell content in each patient from the high and low CALB2 groups in GSE77953 cohort via CIBERSORT algorithm. **(H)** Analysis of the proportion of immune cells between high and low CALB2 groups in GSE71198 cohort via CIBERSORT algorithm. *P < 0.05, **P < 0.01, ****P < 0.0001.

We analyzed the immune infiltration of CALB2 in colorectal cancer with multiple algorithms (**Figure 4A**). Results showed that CALB2 was strongly associated with macrophage, fibroblasts or CAF, and CD8^+^T cell via TIMER, Quantiseq, CIBERSORT, xCell and EPIC algorithms (**Figure 4A**). Correlation analysis strongly suggested a positive correlation between CALB2 and M2 macrophages from TCGA-CRC cohort, GSE77953 and GSE71187 cohorts (**Figures 4B, C**). Similarly, a positive correlation between CALB2 and fibroblasts was also observed in TCGA-CRC, GSE104645 and GSE72970 cohorts (**Figures 4D, E**). A negative correlation between CALB2 and CD8^+^T cells was observed in GSE18105 and GSE35452 cohorts (**Figure 4F**).We chose GSE77953 cohort to analyze the immune infiltration of CALB2 via CIBERSORT algorithm, for CALB2 highly expressed in metastatic colorectal cancer patients (**Figure 1E**), and the analysis results showed that patients with high CALB2 expression had a higher proportion of M2 macrophages and lower proportion of CD8^+^T cells (**Figures 4G, H**).

### CALB2 collaborates with M2 macrophages and CAFs to construct a cold tumor immune microenvironment

**Figure 5:**
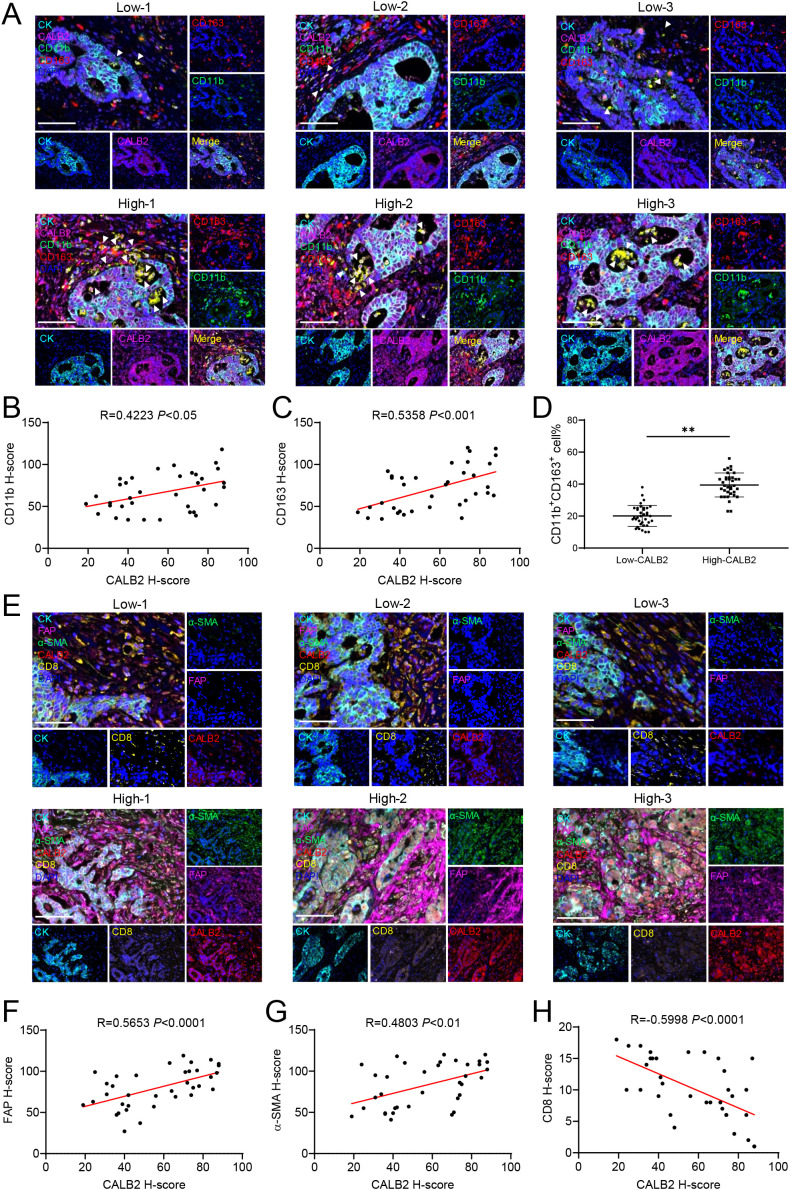
CALB2 collaborates with M2 macrophages and CAFs to construct a cold tumor immune microenvironment. **(A)** Representative multicolor immunofluorescence images of colorectal cancer tissues in the high and low CALB2 groups. The cell nuclei are labeled by DAPI (blue), and the macrophages are labeled by anti-CD163 antibody (red) and CD11b antibody (green). CALB2 is labeled by anti-CALB2 antibody (pink), tumor epithelial cells are labeled by anti-CK antibody (cyan), and white arrows indicate a cluster of CD11b^+^CD163^+^ M2 macrophages in the stroma. Scale: 50μm. **(B)** Correlation analysis of CALB2 H-score and CD11b H-score in all colorectal cancer tissues. **(C)** Correlation analysis of CALB2 H-score and CD163 H-score in all colorectal cancer tissues. **(D)** Proportion analysis of CD11b^+^CD163^+^ labeled M2 macrophages in high and low CALB2 colorectal cancer tissues. **(E)** Representative multicolor immunofluorescence images of colorectal cancer tissues in the high and low CALB2 groups. The cell nucleus is labeled by DAPI (blue), CALB2 is labeled by anti-CALB2 antibody (red), tumor epithelial cells are labeled by anti-CK antibody (cyan), and CD8^+^T cells are labeled by anti-CD8 antibody (yellow). Cancer-associated fibroblasts (CAF) are labeled with anti-α-SMA antibody (green) and anti-FAP antibody (pink), Scale: 50μm. **(F)** Correlation analysis of CALB2 H-score and CD8 H-score in all colorectal cancer tissues. **(G)** Correlation analysis of CALB2 H-score and FAP H-score in all colorectal cancer tissues. **(H)** Correlation analysis of CALB2 H-score and α-SMA H-score in all colorectal cancer tissues. **P < 0.01.

Based on the bioinformation analysis from **Figure 4**, we explored the expression of M2 macrophage, cancer-associated fibroblast and CD8^+^T cell in CRC tumor tissues microenvironment via mIHC. In first panel, we labeled CD11b^+^CD163^+^ cells as M2 macrophages (14) (**Figure 5A**). The results demonstrated that patients with high expression of CALB2 had more M2 macrophages in microenvironment while there were significantly fewer M2 macrophages infiltrating in patients with low CALB2 expression (**Figure 5A**). We analyzed their H-score, results indicated that CALB2 was positively correlated with CD11b and CD163 (**Figures 5B, C**), meanwhile, the percent of CD11b^+^CD163^+^ cells in high CALB2 expression patients was higher than that in low CALB2 expression patients (**Figure 5D**). In the second panel, we marked FAP^+^ or α-SMA^+^ cells as cancer-associated fibroblast and labeled CD8 as CD8^+^T cells (15) (**Figure 5E**). The results demonstrated that patients with high CALB2 expression had more cancer-associated fibroblasts and fewer CD8^+^T cells in microenvironment while there was significantly fewer CAFs and more CD8^+^T cells infiltrating in patients with low CALB2 expression (**Figure 5E**). H-score analysis showed that CALB2 was positively correlated with FAP and α-SMA (**Figures 5F, G**). Additionally, H-score analysis also revealed that CALB2 was negatively correlated with CD8 (**Figure 5H**). In summary, CAFs collaborated with M2 macrophages to construct a cold tumor immune microenvironment preventing CD8^+^T cells infiltrating in high CALB2 expression CRC patients.

### CALB2 is positively correlated with immune regulation and tumor progression related pathways

**Figure 6:**
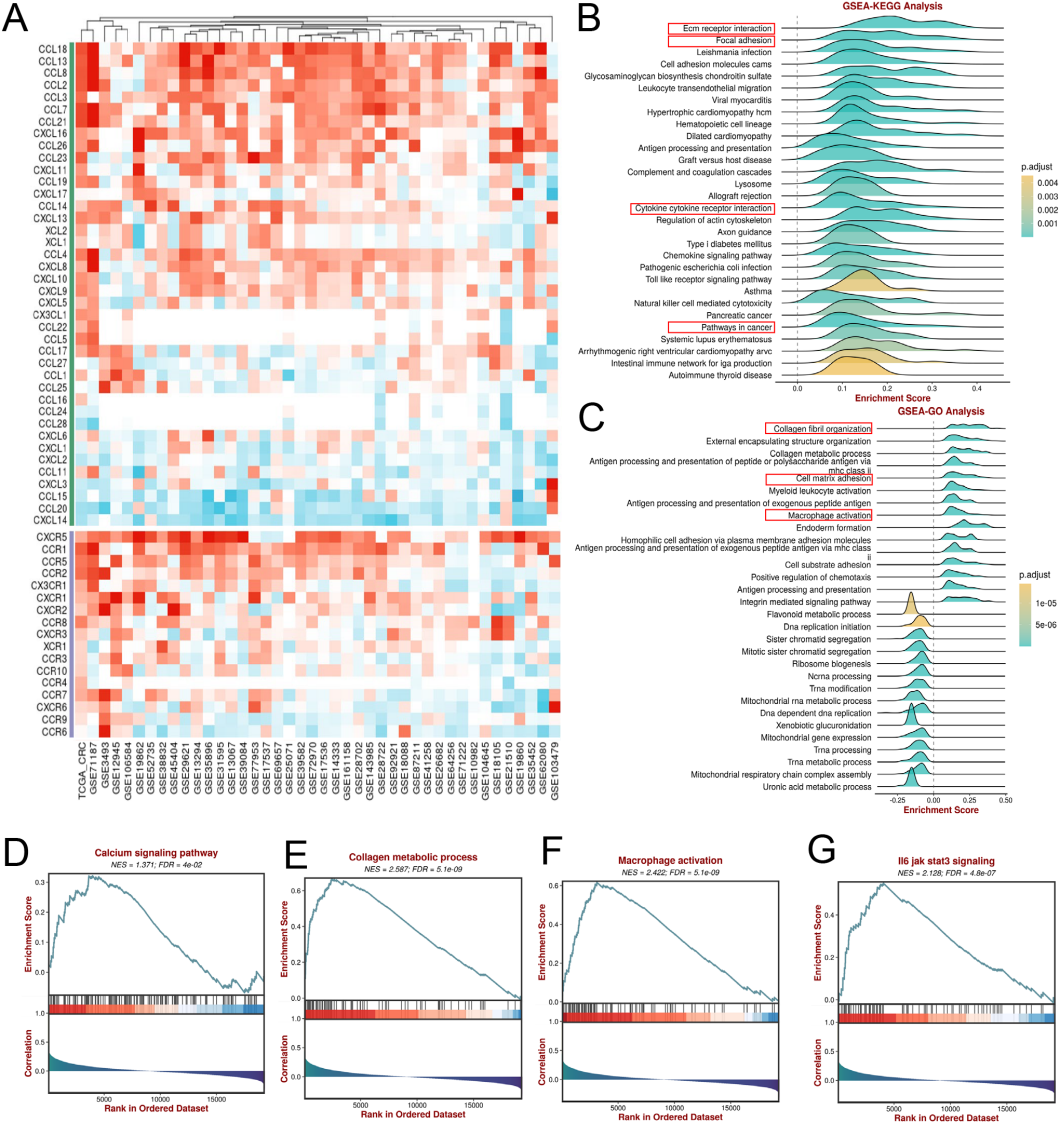
CALB2 is positively correlated with immune regulation and tumor progression related pathways. **(A)** Heat map of CALB2 association with inflammatory chemokines and receptors in TCGA and GEO cohorts. **(B)** Enrichment map of CALB2 GSEA-KEGG analysis in the TCGA-CRC cohort. **(C)** Enrichment map of CALB2 GSEA-GO analysis in the TCGA-CRC cohort. **(D)** Correlation analysis of CALB2 and calcium signaling pathway enrichment in TCGA-CRC cohort. **(E)** Correlation analysis of CALB2 and collagen metabolic process enrichment in TCGA-CRC cohort. **(F)** Correlation analysis of CALB2 and macrophage activation enrichment in TCGA-CRC cohort. **(G)** Correlation analysis of CALB2 and JAK STAT3 signaling pathway enrichment in TCGA-CRC cohort.

Inflammatory chemokines play a variety of roles in tumor immune microenvironment, such as recruitment and guidance. We analyzed the correlation between CALB2 and chemokines in colorectal cancer. Results demonstrated that CALB2 had strong relation with chemokines, like CCL18, CCL13, CCL8 and others (**Figure 6A**). Chemokines not only reshape the immunosuppressive microenvironment by recruiting and inducing macrophages polarization (16), but also act on fibroblasts to promote their activation to CAFs and synergistically drive tumor progression (17). We also perform KEGG analysis and GSEA-GO analysis based on CALB2 expression from TCGA-CRC data. It showed that high CALB2 expression was significantly enriched in ECM receptor interaction, Cytokine receptor interaction, epithelial mesenchymal transition (**Figure 6B**; **Supplementary Figure S1G**), Collagen fibril organization, and Macrophage activation (**Figure 6C**), indicating a strong correlation between CALB2 and tumor progression as well as cellular immunity. Further analysis of pathway enrichment revealed that CALB2, as a calcium-binding protein, is positively correlated with the calcium signaling pathway (**Figure 6D**). CALB2 is also enriched in the Collagen metabolic pathway, suggesting its association with tumor metastasis (**Figure 6E**). Pathway enrichment analysis also revealed that CALB2 was associated with macrophage activation, JAK-STAT3 and the VEGF signaling pathways, suggesting an indispensable role of CALB2 in the immune microenvironment (**Figures 6F, G**; **Supplementary Figure 1H**).

### CALB2 promotes M2 macrophage polarization and inhibits CD8^+^T cells

**Figure 7:**
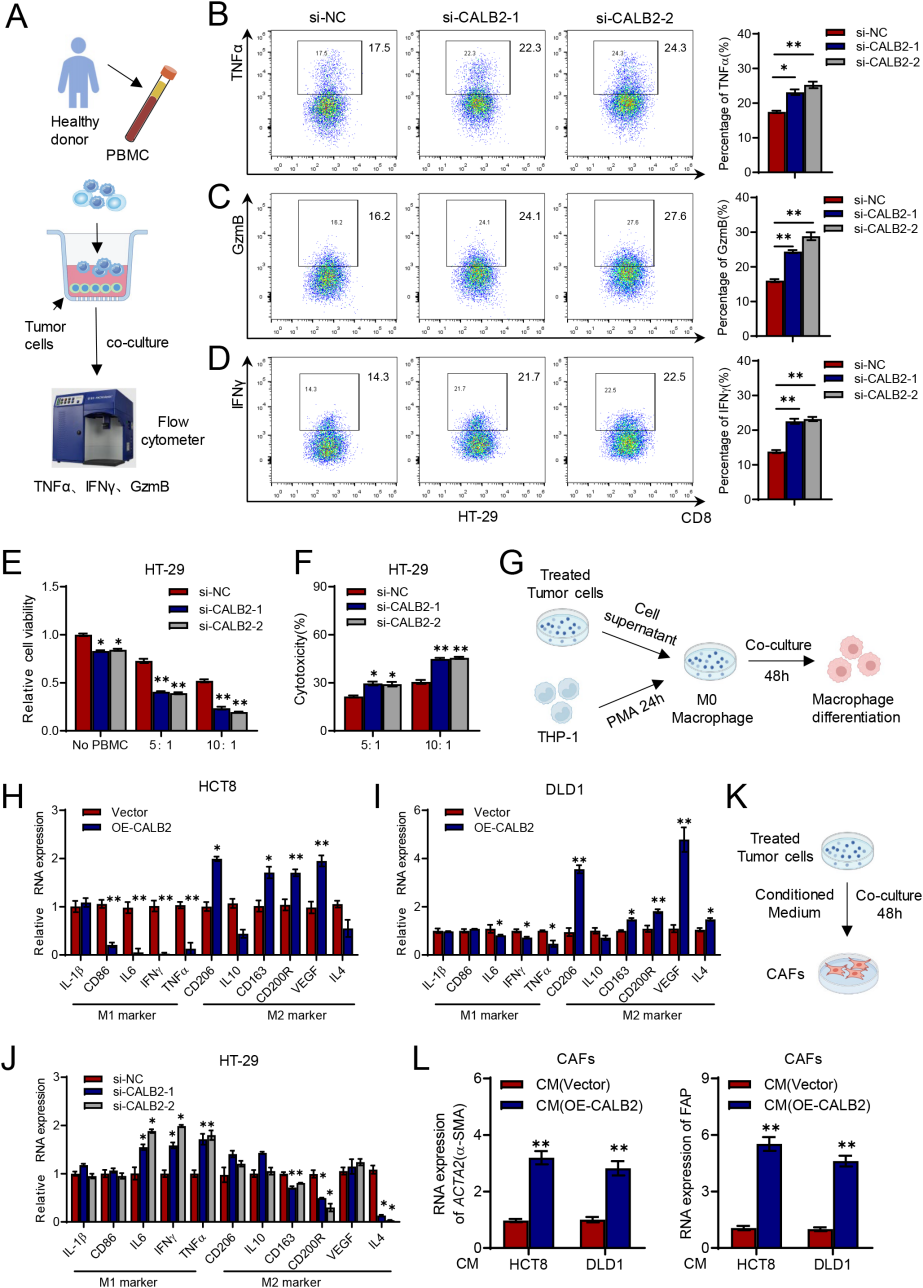
CALB2 promotes M2 macrophage polarization and inhibits CD8^+^T cells. **(A)** Schematic diagram of co-culture of tumor cells and PBMCs. **(B)** The proportion of TNFα was detected by flow cytometry. **(C)** The proportion of GzmB was detected by flow cytometry. **(D)** The proportion of IFNγ was detected by flow cytometry. **(E)** HT-29 cells transfected with si-NC or si-CALB2 were co-cultured with or without PBMCs for 24 h at different effector-to-target (E: T) ratios. Cell viability was normalized to no PBMC controls. **(F)** HT-29 cells transfected with si-NC or si-CALB2 were co-cultured with PBMCs at indicated E:T ratios for 24 h, and cytotoxicity was measured by LDH release assay. **(G)** Schematic diagram of co-culture of tumor cell supernatant and macrophage. **(H)** The expression of M1 and M2 phenotype markers in macrophages was determined by RT-qPCR after co-culture with the indicated HCT8 cells. **(I)** The expression of M1 and M2 phenotype markers in macrophages was determined by RT-qPCR after co-culture with the indicated DLD1 cells. **(J)** The expression of M1 and M2 phenotype markers in macrophages was determined by RT-qPCR after co-culture with the indicated HT-29 cells. **(K)** Schematic diagram of co-culture of tumor conditioned medium and CAFs. **(L)** Biomarkers of CAFs were determined by RT-qPCR after co-culture with the indicated HCT8 and DLD1 cells. *P < 0.05, **P < 0.01.

Immune infiltration analysis in **Figures 4**, **5** showed CALB2 had strong relationship with M2 macrophage, CD8^+^T cell and CAFs, thus we carried co-culture experiments to explore CALB2 effects in immune cells. We isolated peripheral blood mononuclear cell from healthy donors. The treated tumor cells and pre-activated PBMCs were co-cultured for 24–48 hours, and then the secretion factors from CD8^+^T cells were detected by flow cytometry (**Figure 7A**). Results revealed that the percentage of TNFα (**Figure 7B**), GzmB (**Figure 7C**) and IFNγ (**Figure 7D**) were all increased when HT-29 cell was transfected si-CALB2. We also performed additional killing assays. HT-29 cells with CALB2 knockdown were co-cultured with activated T cells, and cytotoxicity was assessed by CCK8 and LDH release assay. As shown in **Figure 7E**, the results of cell viability detection indicated that, compared with the control group, CALB2 knockdown significantly enhanced the cytotoxic effect of T cells on tumor cells, and this cytotoxic effect was particularly evident when the ratio of effector cells to target cells was 10:1. Similarly, LDH release assay results also showed that T cell killing ability was enhanced after CALB2 knockdown (**Figure 7F**).

THP-1 cell was induced to M0 macrophage by PMA and then M0 macrophage was also co-cultured with cell supernatant from tumor cells (**Figure 7G**). We extracted macrophage RNA and results showed M1 macrophage markers like CD86, IL6, IFNγ and TNFα were decreased and M2 macrophage markers like CD163, CD206, CD200R and VEGF were increased when over expressing CALB2 in HCT8 cells (**Figure 7H**). Similarly, M1 macrophage markers like IL6, IFNγ and TNFα were decreased and M2 macrophage markers like CD206, CD163, CD200R, VEGF and IL4 were increased when over expressing CALB2 in DLD1 cells (**Figure 7I**). M1 macrophage markers like IL6, IFNγ and TNFα were increased, M2 macrophage markers like CD163, CD200R and IL4 were decreased when silencing CALB2 in HT-29 cells (**Figure 7J**). Our team previously extracted CAF from colorectal cancer tissues for research (18), thus we conducted a co-culture experiment of tumor cell conditioned medium with CAF to detect the activation of CAFs (**Figure 7K**). *ACTA2* gene, encoding α-SMA protein, and FAP are common markers of CAF activation (19). We extracted CAF RNA and results showed that after co-culturing with the tumor medium overexpressing CALB2 from HCT8 and DLD1 cells, *ACTA2* and FAP both increased, suggesting that CAFs were activated (**Figure 7L**).

### CALB2 secretes CCL5 through STAT3 pathway to promote M2 macrophage polarization and CAF activation

**Figure 8:**
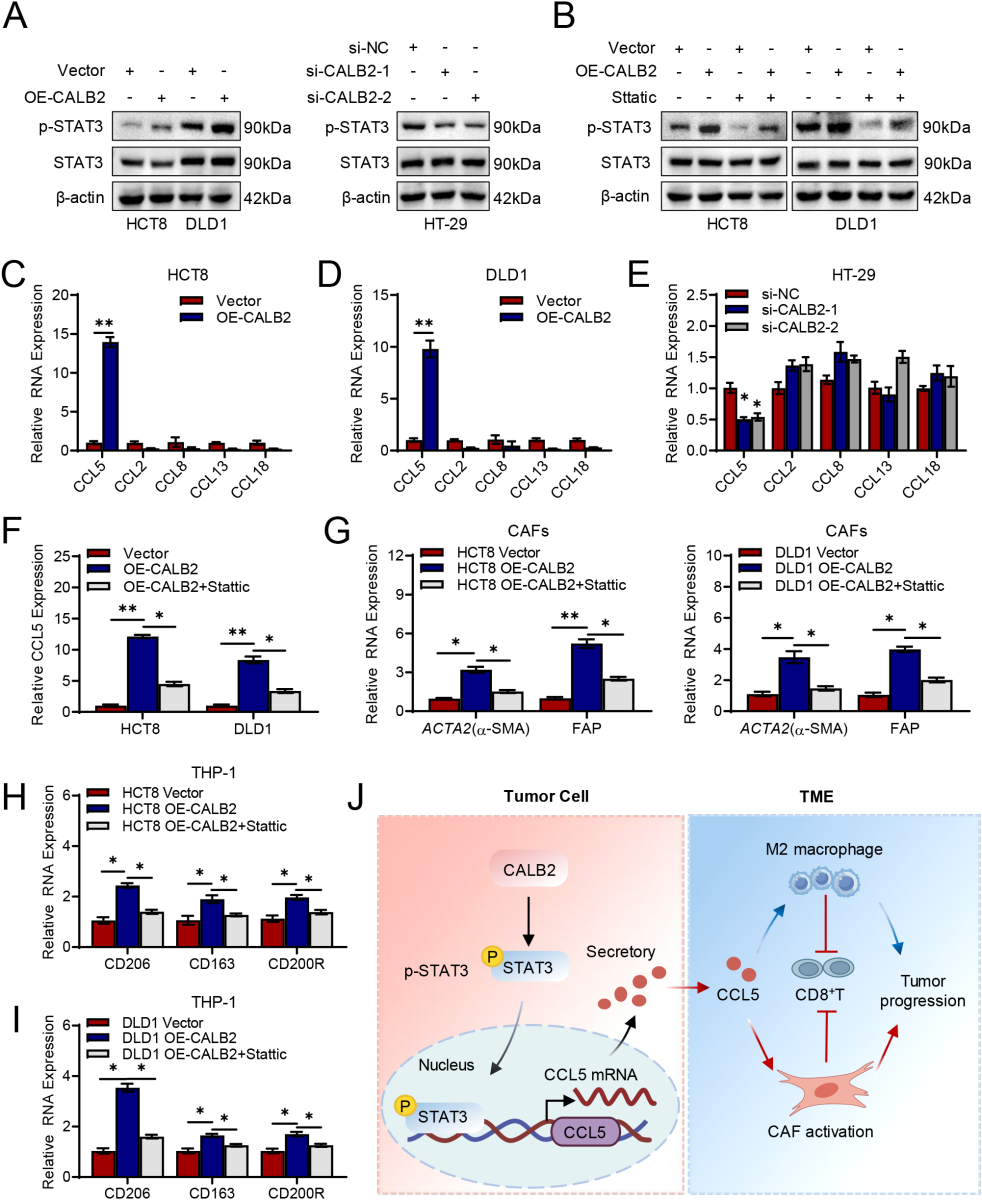
CALB2 secretes CCL5 through STAT3 pathway to promote M2 macrophage polarization and CAF activation. **(A)** Immunoblotting (IB) analysis of STAT3 and p-STAT3 in HCT8, DLD1and HT-29 cells transfected with CALB2 plasmid or si-CALB2. **(B)** Immunoblotting (IB) analysis of STAT3 and p-STAT3 in indicated HCT8 and DLD1 cells added with 10 μM Sttatic for 24h. **(C)** The mRNA levels of chemokines (CCL5, CCL2, CCL8, CCL13 and CCL18) in HCT8 cells transfected with CALB2 plasmid were detected by RT-qPCR. **(D)** The mRNA levels of chemokines (CCL5, CCL2, CCL8, CCL13 and CCL18) in DLD1 cells transfected with CALB2 plasmid were detected by RT-qPCR. **(E)** The mRNA levels of chemokines (CCL5, CCL2, CCL8, CCL13 and CCL18) in HT-29 cells transfected with si-CALB2 were detected by RT-qPCR. **(F)** Expression of CCL5 in HCT8 and DLD1cells added with 10 μM Sttatic for 24h were detected by RT-qPCR. **(G)** Biomarkers of CAFs were determined by RT-qPCR after co-culture with the indicated HCT8 and DLD1 cells. **(H)** Biomarkers of M2 macrophages were determined by RT-qPCR after co-culture with the indicated HCT8 cells. **(I)** Biomarkers of M2 macrophages were determined by RT-qPCR after co-culture with the indicated DLD1 cells. **(J)** Schematic diagram of CALB2 secretes CCL5 through STAT3 pathway to promote M2 macrophage polarization and CAF activation. *P < 0.05, **P < 0.01.

Pathway enrichment analysis in **Figure 6** predicted CALB2 was related with JAK-STAT3 pathway, therefore we detected STAT3 and phosphorylated STAT3 (p- STAT3) in HCT8, DLD1 and HT-29 cells via western blot. Results showed phosphorylated STAT3 is enhanced when overexpression CALB2 in HCT8 and DLD1 cells, meanwhile phosphorylated STAT3 is decreased when silencing CALB2 in HT-29 cells (**Figure 8A**). After the addition of STAT3 inhibitor Stattic (20), results showed that p-STAT3 was reduced in HCT8 and DLD1 compared with the CALB2 overexpression group, suggesting that the effect of CALB2 depends on STAT3 pathway (**Figure 8B**).

Next, we detected several chemokines related with M2 polarization and CAF activation predicted in **Figure 6A**, including CCL5 (21), CCL2 (22), CCL8 (23), CCL13 (24) and CCL18 (25) via RT-qPCR. These chemokines have all been reported to be capable of promoting M2 polarization or CAF activation. The results showed that CCL5 was most significantly increased when overexpressing CALB2 in HCT8 and DLD1 cells (**Figures 8C, D**), meanwhile, CCL5 decreased when silencing CALB2 inHT-29 cells (**Figure 8E**). Therefore, we hypothesized that CALB2 promotes M2 polarization and CAF activation through CCL5 secretion via STAT3 pathway. RT-qPCR results showed CCL2 was reduced when adding STAT3 inhibitor Stattic in CALB2 overexpression group in HCT8 and DLD1 cells (**Figure 8F**). Next, we co-cultured tumor cell supernatants with fibroblasts or macrophages to detect changes in CAF or macrophage markers by RT-qPCR. Treatment of HCT8 and DLD-1 cells with STAT3 inhibitors significantly reduced the ability of their supernatants to promote *ACTA2* (α-SMA) and FAP expression in CAFs (**Figure 8G**). Similarly, M2 macrophage markers including CD206, CD163 and CD200R all reduced when coculturing with supernatants from HCT8 and DLD-1 cells added STAT3 inhibitor (**Figures 8H, I**). These replenishment experiments demonstrate that CALB2 promotes CCL5 secretion through the STAT3 pathway, leading to M2 polarization and CAF activation. In summary, our results revealed CALB2 promotes CCL5 secretion through STAT3 pathway, causing M2 polarization and CAF activation, shaping the immunosuppressive microenvironment and promoting the progression of colorectal cancer (**Figure 8J**).

## Discussion

CRC is the leading cancer-related death and is also the most common malignant tumor worldwide (2). In the current study, we found that CRC patients with high expression of CALB2 had poor prognosis than those with low expression of CALB2. Silencing CALB2 inhibited the proliferation and migration of CRC cells, promoted cell apoptosis, enhanced the killing ability of CD8^+^T cells and inhibited M2 macrophage polarization. Our results indicated that CALB2, as an oncogene in CRC, was associated with the suppressive immune microenvironment and was expected to become the new biomarker for diagnosis and treatment of CRC.

The CALB2 gene encodes a protein called Calbindin2. This is an important calcium-binding protein that acts as a calcium ion buffer and signal sensor within cells. Widely expressed in the central and peripheral nervous systems (26), it participates in regulating the release of neurotransmitters, neuronal excitability, and protecting neurons from toxic effects caused by calcium ions overload by adjusting the concentration of calcium ions within cells (12, 27), which is crucial for maintaining normal neural function. In addition, it is also expressed in many non-neural tissues such as the kidneys and reproductive system, playing a tissue-specific role in calcium signal regulation. It is widely used as an immunohistochemical marker to identify specific types of neurons and diagnose related neurological tumors. Therefore, the role of CALB2 in tumors has received increasing attention. Previous researches have revealed that CALB2 plays an indispensable role in the progression of various tumors. Especially, CALB2 promotes the development of ovarian cancer, and low expression of CALB2 has a better prognosis (28). There are also many studies on CALB2 in digestive tract tumors. In pancreatic cancer, CALB2 is highly expressed in CAF, shaping an inhibitory immune microenvironment to promote the metastasis of pancreatic cancer (29). In liver cancer, CALB2 promotes the epithelial-mesenchymal transition of tumors (10). Consistent with these findings, our research indicates that CALB2 acts as an oncogene in colorectal cancer and it is closely related to the immune microenvironment.

The inhibitory immune microenvironment is one of the key mechanisms by which tumors achieve immune escape. Various immune cells and their secreted factors jointly form a powerful immunosuppressive network. Myeloid-derived inhibitory cells (MDSCs) and M2 macrophages directly inhibit T cell function by secreting inhibitory cytokines like TGF-β and IL-10 (6, 9, 30). In addition, tumor-associated fibroblasts prevent immune cell infiltration by building physical barriers and secreting chemical factors (8), leading to the exhaustion of CD8^+^T cell function and preventing them from effectively recognizing and eliminating tumor cells (31). Our research results indicate that CALB2 is positively correlated with the infiltration of M2 macrophage and CAF, and CALB2 is negatively associated with the CD8^+^T cell infiltration as well. Vitro experiments have also confirmed that CALB2 suppresses CD8^+^T cell cytotoxic ability and promotes M2 macrophage polarization.

STAT3 is a pivotal transcription factor regulating both macrophage polarization and CAF activation within the tumor microenvironment (32). STAT3 signaling promotes immunosuppressive M2 macrophage polarization while suppressing the M1 phenotype (33). Concurrently, STAT3 activation in CAFs drives their pro-tumorigenic functions, creating a feed-forward loop that sustains tumor progression (34). CCL5, a CC motif chemokine, exerts potent immunomodulatory functions through its receptor CCR5 (35). CCL5 induces M2 polarization of tumor-associated macrophages and promotes the transformation of fibroblasts into CAFs, and has been reported to be regulated by STAT3 (36, 37). In the present study, we identified a novel mechanism by which CALB2 activates the STAT3 signaling pathway in tumor cells, leading to enhanced CCL5 secretion. This CALB2-CCL5 axis subsequently induces M2 macrophage polarization and promotes fibroblast activation. Our findings establish CALB2 as an upstream regulator of the STAT3/CCL5 axis, highlighting its critical role in orchestrating tumor-stromal interactions and remodeling microenvironment.

## Conclusion

Our investigation revealed that high expression of CALB2 was associated with poor prognosis in CRC patients. Knockdown of CALB2 suppressed colorectal cancer via inhibiting cell proliferation, migration and increasing apoptosis. Tumors exhibiting high CALB2 expression were characterized by an immunosuppressive microenvironment, marked by an elevated infiltration of M2 macrophages and cancer-associated fibroblasts, alongside a reduced presence of CD8^+^T cells. CALB2 promoted CCL5 secretion via STAT3 pathway, facilitating M2 polarization and CAF activation. Our study demonstrates that CALB2 has the potential to predict the prognosis of colorectal cancer and can be used as a therapeutic target for colorectal cancer.

## Data Availability

The original contributions presented in the study are included in the article/**Supplementary Material**. Further inquiries can be directed to the corresponding author.

## Supplementary material

Supplementary Figure S1 **(A)** The expression of CALB2 in different primary tumor stages of the GSE41258 cohort. **(B)** The expression of CALB2 in different clinical stages of the GSE38832 cohort. **(C)** Overall survival analysis of colorectal cancer patients with high and low CALB2 expression from GSE17537 cohort. **(D)** Disease free survival analysis of colorectal cancer patients with high and low CALB2 expression from TCGA-CRC cohort. **(E)** Progress-free survival analysis of colorectal cancer patients with high and low CALB2 expression from TCGA-CRC cohort. F The viability of HT-29 cells transfected with si-CALB2–2 was detected by clone formation assay. G Enrichment map of CALB2 GSEA-Hallmark analysis in the TCGA-CRC cohort. H Correlation analysis of CALB2 and VEGF signaling pathway in TCGA-CRC cohort.
