# Supplementary figures and images for: CALB2 mediates tumor progression and immune escape in colorectal cancer by shaping an inhibitory immune microenvironment
Source: Front Immunol. 2026 Apr 15;17:1791363. doi: 10.3389/fimmu.2026.1791363 (PMC13124944; doi:10.3389/fimmu.2026.1791363)

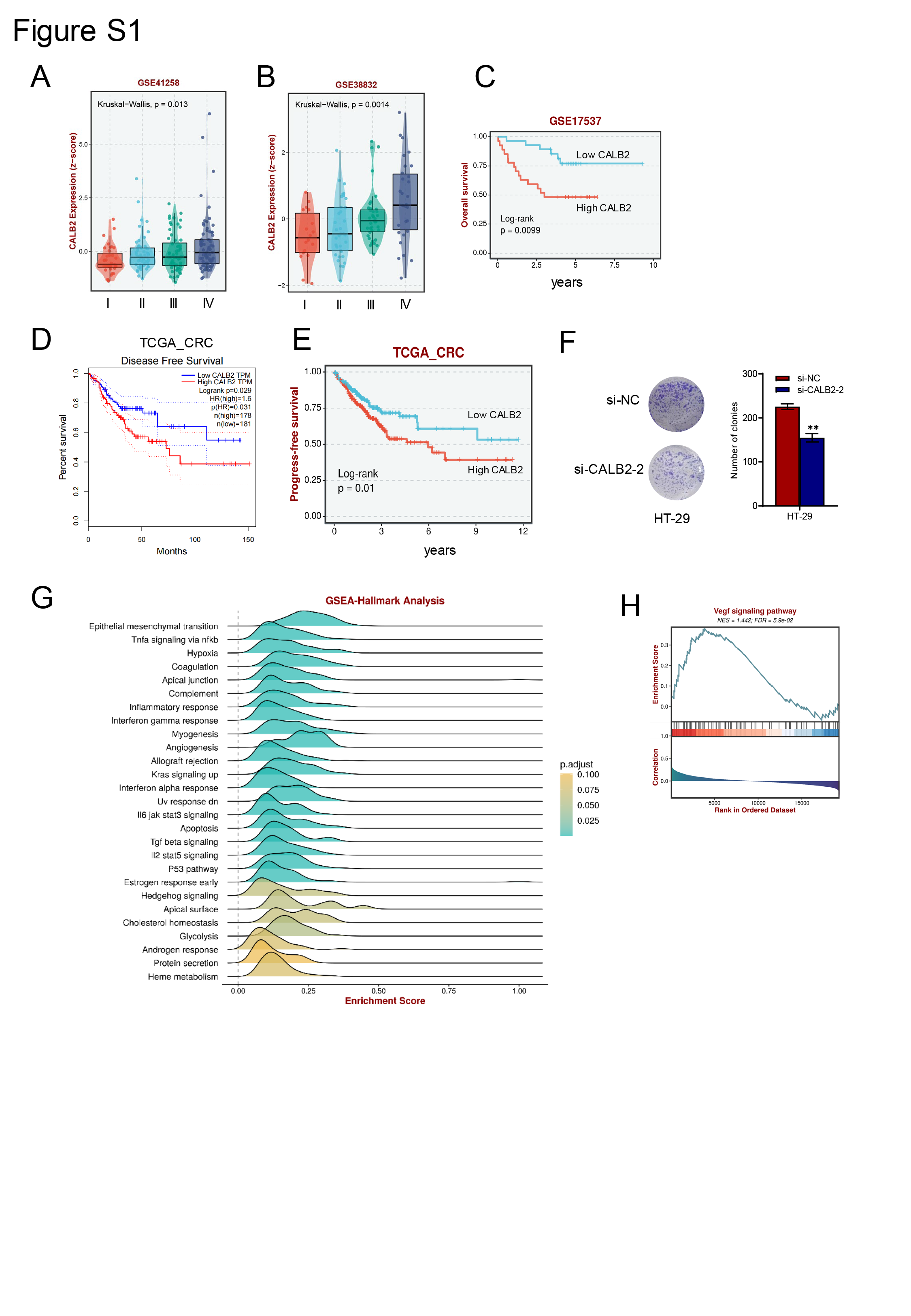

Supplement: Supplementary Figure S1 — (A) The expression of CALB2 in different primary tumor stages of the GSE41258 cohort. (B) The expression of CALB2 in different clinical stages of the GSE38832 cohort. (C) Overall survival analysis of colorectal cancer patients with high and low CALB2 expression from GSE17537 cohort. (D) Disease free survival analysis of colorectal cancer patients with high and low CALB2 expression from TCGA-CRC cohort. (E) Progress-free survival analysis of colorectal cancer patients with high and low CALB2 expression from TCGA-CRC cohort. F The viability of HT-29 cells transfected with si-CALB2–2 was detected by clone formation assay. G Enrichment map of CALB2 GSEA-Hallmark analysis in the TCGA-CRC cohort. H Correlation analysis of CALB2 and VEGF signaling pathway in TCGA-CRC cohort. [file Image1.tif]
